# Storage Duration of Red Blood Cell Transfusion and *Clostridium difficile* Infection: A Within Person Comparison

**DOI:** 10.1371/journal.pone.0089332

**Published:** 2014-02-21

**Authors:** Mary A. M. Rogers, Dejan Micic, Neil Blumberg, Vincent B. Young, David M. Aronoff

**Affiliations:** 1 Department of Internal Medicine, University of Michigan, Ann Arbor, Michigan, United States of America; 2 Department of Pathology and Laboratory Medicine, University of Rochester Medical Center, Rochester, New York, United States of America; 3 Department of Microbiology and Immunology, University of Michigan, Ann Arbor, Michigan, United States of America; 4 Department of Medicine, Vanderbilt University Medical Center, Nashville, Tennessee, United States of America; University of Arizona, United States of America

## Abstract

**Objective:**

Randomized controlled trials demonstrated that red blood cell (RBC) transfusion elevates the risk of infection, and trials are underway to evaluate whether RBC storage affects outcomes. We previously reported that transfusion predicts *Clostridium difficile* infection (CDI) and, therefore, planned an investigation to examine this further using a more robust design.

**Design:**

Within-person case-crossover study. Hospitalizations in which CDI developed (n = 406) were compared to hospitalizations for the same individuals in which CDI did not occur (n = 949). Transfusion volume and storage duration were assessed prior to the onset of CDI.

**Setting:**

University of Michigan Health System.

**Patients:**

Participants were individuals with a diagnosis of CDI from July 2009 through June 2012.

**Measurements and Main Results:**

During the hospitalizations when CDI occurred, 34.7% of the patients received allogeneic RBC transfusions (mean volume, 688 ml) compared to 19.0% of patients in hospitalizations without CDI (mean volume, 180 ml). The odds of healthcare-associated CDI increased by 76% (95% CI 1.39–2.23) for every liter of RBCs transfused and was elevated in both nonsurgical (OR = 1.90) and surgical (OR = 1.86) hospitalizations. In patients who received RBC transfusions, the odds of developing CDI increased by 6% for every additional day of RBC stored and by 53% for every week of additional storage (*P* = 0.002).

**Conclusions:**

Hospitalizations in which a patient received a greater volume of RBC transfusions were more likely to be associated with the development of CDI. RBC units stored for a longer duration were associated with the development of healthcare-associated CDI after adjustment for RBC volume.

## Introduction

The Choosing Wisely® initiative from the American Board of Internal Medicine lists the avoidance of red blood cell transfusions for arbitrary thresholds among its list of *Five Things Physicians and Patients Should Question* [1]. This advice coincides with the strong recommendation from AABB (formerly American Association of Blood Banks) to adhere to a restrictive transfusion strategy (7 to 8 g/dL) for hospitalized stable patients, based on a systematic review of randomized clinical trials performed between 1950 and 2011 [2]. Evidence from the meta-analyses demonstrated lower mortality and infection in patients undergoing a restrictive transfusion strategy compared to those receiving allogeneic red blood cell (RBC) transfusions under a more liberal strategy [2].

In addition to judicious attention to appropriate use of RBC transfusion, there has been interest in whether the increased risk of infection after RBC transfusion is related to the length of storage for RBC units. The effect of storage on red blood cells has been documented [3]–[5], but the clinical consequences of receiving transfusions of longer storage duration are currently a subject of debate. There are several randomized controlled trials underway designed to assess patient outcomes with varying RBC storage lengths for patients undergoing cardiac surgery (Red Cell Storage Duration Study or RECESS [6], Red Cell Storage Duration and Outcomes in Cardiac Surgery trial [7]), and in critically ill patients (Age of Blood Evaluation trial or ABLE [8]). Preliminary pilot randomized trials have not shown significant differences in clinical outcomes for length of storage in critically ill or surgical patients [9]–[11] or for major neonatal morbidities or infectious complications in infants [12].

Allogeneic RBC transfusion has been associated with infection in two broad respects – one with the inadvertent contamination of blood products with infectious agents [13] and the other through antigenic challenge resulting in host immunomodulation and elevated rates of infection [14], [15]. While post-transfusion infection has been reported for various sites, we had previously shown an association between transfusion and infection with *Clostridium difficile* (odds ratio (OR), 3.16) in patients undergoing coronary artery bypass graft surgery [16]. These findings were similar to those found by Crabtree *et al.* [17] in cardiac surgery patients (OR, 3.28) and in a large prospective study [18] at 10 centers (relative risk, 2.1). With these findings in mind, we designed a within-person comparative study to assess whether RBC transfusion and the storage duration of the RBC units were associated with the development of *C. difficile* infection (CDI) in a hospital setting. This within-person study design has distinct advantages compared to trials with concurrent controls. First, a within-person comparison provides a better match of host genetic factors, health-related behaviors, and chronic comorbidities compared to between-person comparisons. Second, within-person comparison studies often can provide information on a wider range of patients without restrictive entry criteria [19].

## Materials and Methods

### Setting and Ethics Approval

The University of Michigan Health System (UMHS) includes a 930-bed tertiary care inpatient facility. The health system utilizes a comprehensive electronic medical record system and electronic file server that provides access to patient records. This study was approved by the University of Michigan Institutional Review Board. Written informed consent from study participants (and guardians for minors) was obtained for those who submitted stool samples. The IRB exempted informed consent for the hospitalized patients in which only retrospective database information was utilized.

### Study Design

A within-person case-crossover design was utilized, whereby all patients with healthcare-associated CDI were initially identified from July 1, 2009 through June 30, 2012. This constituted the index hospitalization. For each index hospitalization, subsequent hospitalizations during the same time frame in which no *C. difficile* was isolated served as the control or comparator hospitalizations. Events that occurred prior to the positive stool assay for *C. difficile* during the index hospitalization were compared to events that occurred during the hospitalizations in which the infection was not isolated (comparator hospitalizations). The question being answered was, “Why did the patient develop CDI during the index hospitalization but not during their subsequent hospitalizations?”.

All hospitalizations with positive assays for *C. difficile* as documented by the Clinical Microbiology Laboratory (July 1, 2009 through June 30, 2012) were identified. Initial sample testing was performed at the discretion of the inpatient care teams. Stool samples were transported to the Clinical Microbiology Laboratory in Cary-Blair transport medium. The C. DIFF QUIK CHEK COMPLETE® test (Techlab, Inc., Blacksburg, VA) for *C. difficile* glutamate dehydrogenase (GDH) and toxins A or B by enzyme immunoassay (EIA) was performed according to standard protocols. All GDH+/toxin− stool tests were subjected to analysis for the *tcdB* gene by real-time PCR (BD GeneOhm™ Cdiff Assay; Franklin Lakes, NJ). A positive result consisted of GDH+/toxin+ by EIA or GDH+/toxin+ confirmed by real-time PCR.

Healthcare-associated CDI was of primary interest, so exclusion criteria included: (a) patients whose reason for admission was CDI (principal diagnosis, ICD-9 code 008.45); and (b) patients with a stool sample collected in the first 48 hours of admission which was positive for *C. difficile*.

Blood transfusions administered during the study period were performed at the discretion of the inpatient care teams according to standard protocols. Allogeneic blood products were obtained from the University of Michigan Blood Bank and Transfusion Service. Both red cell components and platelets were leukocyte-reduced through prestorage filtration. Data regarding component types, dispense dates, expiration dates, and volume per unit were available for purposes of this study. Data were also extracted from the electronic medical record regarding patient characteristics (age at the time of admission, admission date, discharge diagnoses (principal and secondary), gastrointestinal surgery (invasive procedures as well as endoscopy), cardiovascular surgery (including cardiac catheterization), transplantation, and dialysis. For purposes of this investigation, hospitalization with “surgical procedures” refers to those in which patients underwent gastrointestinal surgery, cardiovascular surgery, and/or transplantation. Detailed information was also available regarding medications, including dates and timing of administration as well as dosages. Drugs of interest were those known to be related to CDI (e.g., antibiotics, proton pump inhibitors) and those related to immunomodulation (e.g., immunosuppressants, statins). All exposures were censored on the day prior to stool collection for those patients with CDI. Data regarding medications, procedures and transfusions were known for all patients during hospitalization and therefore, there were no missing values.

### Data Analysis

Data analyses began with descriptive statistics of the patients who developed healthcare-associated CDI. This was followed by examination of the frequencies of transfusion, medications and procedures during the index and comparator hospitalizations. Mean, median, maximum and cumulative volumes of RBC and platelet transfusions were determined. To account for the within-person matching, a conditional logit model was used to generate odds ratios (OR) and 95% confidence intervals (CI) for the association between exposures and CDI (outcome). This was offset by the natural log of the time (days) at risk for CDI (i.e., length of time from admission to stool collection for positive *C. difficile* for the index hospitalization, and length of hospital stay for comparator hospitalizations). The interpretation of the OR reflects the odds of developing CDI during a hospitalization relative to other hospitalizations for the same patient (reference category).

Analyses were conducted to examine whether the age of the RBC transfusion was related to the probability of developing CDI. These analyses were restricted to only those matched hospitalizations in which RBC transfusions occurred (in both the index and comparator hospitalizations). In a conditional logit model, RBC storage times (days) were modeled in several ways; the mean storage time of the units given during the hospitalization (prior to CDI), the median storage time, and the maximum storage time were calculated. In addition to days of storage, odds ratios were calculated for weeks of RBC storage. We also conducted an analysis whereby the age of each blood unit was retained in the model, accounting for the repeated units given to each patient over time. To do this, we used multilevel mixed-effects logistic regression allowing for a random intercept for each patient. The number of days of storage was regressed on CDI (index versus comparator hospitalizations), as were the number of weeks of storage. In this 2-level model, repeated administration of RBC units was nested within each patient. Statistical analyses were performed by using Stata/MP 12.1 (StataCorp LP, College Station, TX, USA). Alpha was set at 0.05, 2-tailed.

## Results

During the study period, 406 patients were identified with healthcare-associated CDI (index hospitalizations) and these were matched to 949 comparator hospitalizations (without isolation of CDI). The patients were equally divided by gender (Table 1) and encompassed a wide age range, from 3 months to 95 years of age. Slightly more than half (54.5%) of the index admissions were considered emergent while 63.6% (604/949) of the comparator hospitalizations were emergent admissions.

**Table 1:** Characteristics of Patients with Healthcare-Associated *Clostridium difficile* Infection.

Characteristic	Number	Percent
Age (years):		
<20	46	11.3
20–29	28	6.9
30–39	30	7.4
40–49	62	15.3
50–59	85	20.9
60–69	85	20.9
≥70	70	17.2
Gender:		
Males	207	51.0
Females	199	49.0
Race:		
Caucasian	323	79.6
African-American	64	15.8
Other	19	4.7
Index admission:		
Scheduled	111	27.3
Urgent	74	18.2
Emergent	221	54.4
TOTAL	406	100.0

For the index hospitalizations, the median number of days from admission to a positive stool test was 5 days (interquartile range, 3 to 10 days) and the mean number of days was 8.6 (SD, 10.8). For the comparator hospitalizations, the median number of days from admission to discharge was 5 days (interquartile range, 3 to 8 days) and the mean number of days was 6.5 (SD, 6.6).

**Figure 1:**
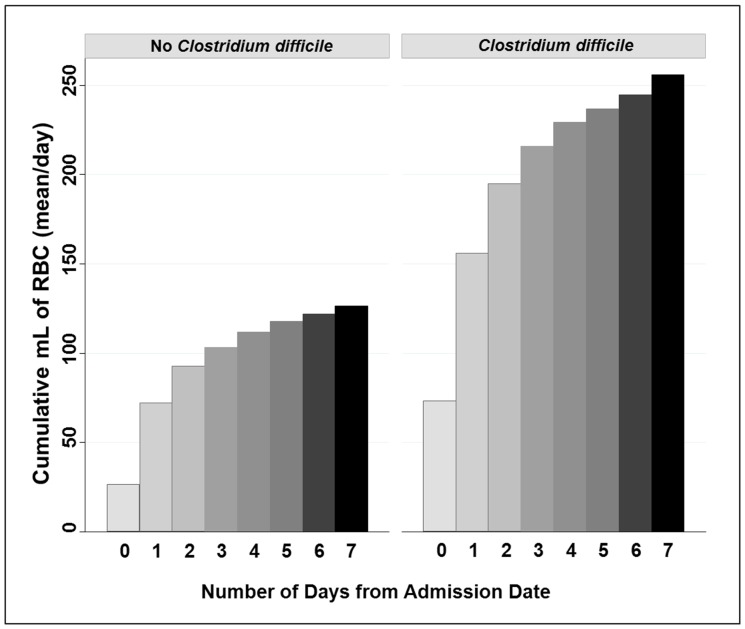
Cumulative Volume of Red Blood Cell Transfusions given during the First Week of Hospitalization Prior to the Development of Healthcare-Associated *Clostridium difficile* Infection and in Comparator Hospitalizations.

RBC transfusions were administered more often during hospitalizations in which CDI occurred. Figure 1 shows the cumulative volume of RBC transfusions given during the first 7 days of hospitalization. Volumes of blood components were censored on the day before the collection of the positive *C. difficile* isolate, therefore only RBC volumes administered prior to the isolation of *C. difficile* are represented. During the hospitalizations in which CDI occurred, 34.7% of the patients received a RBC transfusion (Table 2). For the comparator hospitalizations, 19.0% of the hospitalizations included RBC transfusions. Platelet (PLT) transfusions and plasma transfusions occurred prior to CDI in 14.8% and 12.8% of patients, respectively; the comparator frequencies were 8.3% and 5.4%. Patients transfused with platelets frequently received RBC transfusions as well (82% or 114/139 of the hospitalizations with PLT transfusions also contained RBC transfusions). Likewise, patients transfused with plasma commonly received RBC transfusions (74% or 76/103 of the hospitalizations).

**Table 2:** Exposures prior to Healthcare-Associated *Clostridium difficile* Infection and during Comparator Hospitalizations.

	Hospitalization with *Clostridium difficile* (n = 406)		Comparator Hospitalizations (n = 949)		*P*-values^a^
Exposures	Percent	95% CI	Percent	95% CI	
Red blood cell transfusions	34.7	30.1, 39.4	19.0	16.5, 21.5	<0.001
Platelet transfusions	14.8	11.3, 18.2	8.3	6.6, 10.1	0.004
Plasma transfusions	12.8	9.5, 16.1	5.4	3.9, 6.8	<0.001
Gastrointestinal surgeries	24.1	20.0, 28.3	13.7	11.5, 15.9	<0.001
Cardiovascular surgeries	34.2	29.6, 38.9	14.3	12.1, 16.6	<0.001
Antibiotics	82.8	79.1, 86.4	75.7	72.9, 78.4	0.005
Immunosuppressants	46.8	41.9, 51.7	48.1	44.9, 51.2	0.463
Proton pump inhibitors	60.8	56.1, 65.6	56.3	53.1, 59.4	0.399
Histamine-2 receptor antagonists	26.6	22.3, 30.9	24.0	21.3, 26.7	0.089
Statins	25.4	21.1, 29.6	23.2	20.5, 25.9	0.711
Dialysis	5.4	3.2, 7.6	5.7	4.2, 7.2	0.939
Chemotherapy	0.5	0.0, 1.2	1.3	0.6, 2.0	0.165
Bone marrow transplant	4.2	2.2, 6.1	0.0	0.0, 0.0	–b
Other transplants	6.7	4.2, 9.1	1.3	0.1, 2.0	<0.001

a *P*-values from matched analyses (conditional logit model).

b Unable to calculate.

Gastrointestinal and cardiovascular surgeries were more common in hospitalizations during which CDI occurred than in comparator hospitalizations, as shown in Table 2. CDI was also more likely to occur in patients undergoing transplantation. Antibiotic, immunosuppressant and proton pump inhibitor use was common both in index and comparator hospitalizations (Table 2). Antibiotic use was predictive of healthcare-associated CDI (p = 0.005).

Mean volumes of transfusion are listed for index and comparator hospitalizations (Table 3) showing greater volumes during the hospitalizations in which CDI occurred. Odds ratios listed in Table 3 reflect the change in the odds of CDI with every liter increase in transfusion volume. The odds of CDI increased by 76% for each liter of RBC transfused (OR = 1.76; 95% CI 1.39-2.23). This elevation in the odds of CDI was consistent irrespective of whether RBC transfusions were administered with or without platelets (OR, 1.35 and OR, 1.62 respectively). For hospitalizations in which surgical procedures were not performed, the odds of CDI increased by 90% for every liter increase in RBC transfusion volume. The odds ratio for volume of RBC transfusion was significantly elevated, at 1.86 (95% CI, 1.31–2.65), for hospitalizations in which surgical procedures were performed. These were hospitalizations in which surgical procedures were performed both during the index hospitalization as well as during the comparator hospitalizations for the same person. When analyses were restricted to just those hospitalizations in which cardiovascular surgeries were performed, the odds of developing CDI were 2.84 fold greater for each liter of RBCs administered (95% CI, 1.27–6.36).

**Table 3:** Odds Ratios for the Association between Volume of Transfusions and Healthcare-Associated *Clostridium difficile* Infection.

	Number	Hospitalization with *Clostridium difficile*		Comparator Hospitalizations		Adjusted Odds Ratio		
Characteristics		Mean mL	95% CI	Mean mL	95% CI	OR	95% CI	*P*- value
***All Hospitalizations:*** a								
RBC transfusion	1355	688	520, 855	180	144, 216	1.76	1.39, 2.23	<0.001
***Hospitalizations without Surgical*** ***Procedures:*** b								
RBC transfusion	475	365	132, 598	108	76, 141	1.90	1.02, 3.55	0.044
***Hospitalizations with*** ***Surgical Procedures:*** b								
RBC transfusion	555	932	700, 1164	319	236, 403	1.86	1.31, 2.65	0.001
***Hospitalizations with Cardiovascular*** ***Surgeries:*** b								
RBC transfusion	104	1225	881, 1568	458	296, 620	2.84	1.27, 6.36	0.011

a Odds ratio per RBC liter increase in transfusion adjusted for surgical procedures, chemotherapy, dialysis, and number of doses of antibacterial, immunosuppressant, proton pump inhibitor, histamone-2 receptor antagonist, and statin medications.

b Odds ratio per RBC liter increase in transfusion adjusted for chemotherapy, dialysis, and number of doses of antibacterial, immunosuppressant, proton pump inhibitor, histamone-2 receptor antagonist, and statin medications.

RBC storage was also examined. Duration of storage ranged from 2 to 42 days, with a mean of 25 days and a median of 26 days. For those patients who received RBC transfusions, the number of days the RBCs were stored was regressed on healthcare-associated CDI in a conditional logit model, after adjustment for volume of RBCs received, surgical procedures, and medications (Table 4). There was a significant association between RBC storage age and the development of CDI. When modeled with a conditional logit, both the mean and median storage age were significant, while the maximum age yielded a result that was slightly above alpha (*P* = 0.063). For every additional day that the RBCs were stored, the odds of developing CDI increased by 6% in the multi-level model (*P* = 0.002). For every additional week that the RBCs were stored, the odds of developing CDI increased by 53% (*P* = 0.002). In a sensitivity analysis, the multi-level model with fixed effects only (no random intercept) yielded an odds ratio of 1.05 (95% CI, 1.01–1.09) for days of storage and of 1.40 (95% CI, 1.06–1.85) for weeks of storage.

**Table 4:** Odds Ratios for the Association between Storage Time of Red Blood Cell Transfusions and Development of Healthcare-Associated *Clostridium difficile* Infection.

	Number of Hospitalizations with RBC transfusion	Adjusted Odds Ratio, Days of Storage		Adjusted Odds Ratio, Weeks of Storage	
Characteristics		OR^a^	95% CI	OR^a^	95% CI
***Conditional Logit*** b	321				
Mean RBC unit age		1.08	1.01, 1.15	1.66	1.04, 2.65
Maximum RBC unit age		1.05	1.00, 1.11	1.42	0.98, 2.07
Median RBC unit age		1.07	1.01, 1.14	1.61	1.04, 2.51
***Multi-Level Mixed Effects Logit*** c	321				
RBC unit age		1.06	1.02, 1.10	1.53	1.17, 1.99

a Odds ratio per unit increase in RBC transfusion storage time, adjusted for volume of RBC given, surgical procedures,

chemotherapy, dialysis, and number of doses of antibacterial, immunosuppressant, proton pump inhibitor, histamone-2 receptor antagonist, and statin medications.

b Conditional logistic regression model accounting for matched design, offset by days at risk.

c Random intercept multi-level mixed effects logit model with repeated transfusions nested within individual.

Of the RBC units administered, 37.6% were irradiated. There was no statistical difference between irradiation status (yes/no) of the RBCs and hospitalizations with and without CDI (p = 0.435).

## Discussion

Transfusion volume and duration of RBC storage significantly impacted the likelihood of developing healthcare-associated CDI. The relationship between RBC transfusion and CDI was not due to confounding by patient demographics, host genetic factors, or a past medical history of underlying chronic comorbidities because the comparator hospitalizations were obtained from the same individuals. Moreover, the findings regarding RBC storage duration and the development of healthcare-associated CDI were not due to confounding by indication, since only those who received transfusions (i.e., in whom transfusions were deemed indicated) were included in the analysis.

Patients who developed CDI were more likely to have undergone surgical procedures. However, the association between RBC transfusion and CDI remained after restriction to only those instances in which surgical procedures occurred in both the index hospitalization and the comparator hospitalizations. Our entry criteria were such that all patients who developed healthcare-associated CDI within the 3-year study period were included, reducing the likelihood of selection bias. In addition, the laboratory technicians assessing *C. difficile* assays were blinded to the transfusion status of the patient, which reduces the likelihood of detection bias. Furthermore, the comparator hospitalizations occurred after the index hospitalization so, *a priori*, one might expect a greater risk of CDI during subsequent hospitalizations [20] – not a lower risk. It is interesting to note that the comparator hospitalizations (in which CDI did not occur) were more likely to be emergent admissions (63.6%) compared to the index hospitalization (54.5%) and this suggests that the patient’s condition at the time of admission was not necessarily graver when the healthcare-associated CDI occurred than during the comparator hospitalizations. While it is conceivable that there is some unknown simultaneous factor strongly correlated with RBC storage duration that confers increased risk for the development of CDI, to our knowledge such factor has not been identified in the literature. On the other hand, the evidence for immunomodulatory effects related with storage duration of RBCs has been widely documented [3]–[5].

The association between the volume of RBC transfusions and the development of CDI was dose-dependent. Each additional liter of RBCs transfused increased the odds of CDI by 76%. For hospitalizations in which cardiovascular surgeries were performed, the odds of developing CDI were 2.84 fold greater for each liter of RBCs administered. This is in agreement with our previous study in which we demonstrated a 3.16 fold increased odds of CDI with RBC transfusions in a Medicare population undergoing cardiac surgery [16]. Crabtree *et al.* also reported this association in cardiac surgery patients at two centers between 1997 and 2004 [17]. With a control population matched 3∶1 based on date of surgery and institution, blood product transfusion was associated with a 3.28 fold increased odds of developing CDI in a multivariable model adjusted for baseline patient and operative characteristics [17]. In 2013, results from a prospective study of 5158 adults who were observed for 65 days after cardiac surgery [18] indicated a significantly higher rate of CDI in patients transfused with RBCs (RR = 2.1; 95% CI 1.2–3.7 calculated from data in Table 2).

Evidence for an elevated risk of infection from the use of a liberal blood transfusion strategy comes from a Cochrane meta-analysis of six randomized controlled trials [21]. The summary effect indicated a 19% lower risk of infection when restrictive hemoglobin triggers are used [21]. Underlying reasons for this relationship have been studied in some detail. Stored red cells have demonstrated a wide range of time-dependent changes including metabolic, enzymatic, oxidative and physiologic alterations, affecting both structure and function [3]. Recently, transfusion-related acute gut injury has been described, linking RBC transfusion with necrotizing enterocolitis in the naïve gut [22]. While the underlying mechanisms are still under investigation, it is interesting to note that, in both instances (naïve gut and post-antibiotic gut such as in CDI), there is dramatic flux in the intestinal microbiota, which may have implications for the gut’s normal role in modulating immune cell differentiation [23]. With respect to immunomodulatory effects, RBCs have been shown to suppress monocyte function which is exacerbated by the length of storage and modified by the type of preservative solution [24], [25]. Packed red blood cells also depress peripheral T-cell proliferation in a dose-response manner [26]. Stored RBCs elevate levels of plasma nontransferrin bound iron, encourage tissue deposition of iron, and initiate inflammation which increase inflammatory cytokines and oxidative damage [27]. The clinical syndrome associated with the immune down-regulation following blood product administration has been referred to as transfusion-associated immunomodulation (TRIM). Clinical evidence for TRIM has been recognized in the medical literature for a considerable length of time [28], and our study potentially extends the role of TRIM to healthcare-associated CDI.

Each year, more than 300,000 *C. difficile* infections occur in US hospitals alone, and CDI was reported as the 17^th^ leading cause of death in Americans ages 65 years and older in 2011 [29], [30]. Moreover, infection with *C. difficile* tends to recur, contributing to hospital readmissions; 32.7% of Medicare beneficiaries and 30.2% of Medicaid recipients experience a 30-day readmission after a hospitalization with CDI [31]. The target of a 30% reduction in CDI in hospitals by the end of 2013 set by the National Action Plan for Prevention of Health-Care Associated Infections has not been met [32]. This may be, in part, due to a greater need for initiatives that have been shown to impact clinical CDI outcomes. In a systematic review of prevention strategies to reduce the incidence of CDI, there was moderate evidence of the effectiveness of restrictive antibiotic practices but limited evidence regarding other approaches, such as environmental preventive interventions [33]. Our finding that blood product transfusion may contribute to this problem is significant in that it highlights potential areas for improvement. The hope is that greater attention to blood management programs such as the Choosing Wisely® initiative will reap supplemental benefits for patients.

## Conclusions

In conclusion, we demonstrate dose-dependent associations between both the volume and length of storage for RBC transfusion and the development of healthcare-associated CDI. Our study strengthens the evidence for judicious use of RBC transfusion and the incorporation of evidence-based guidelines in clinical settings requiring of blood product support.
